# Severity of influenza-associated hospitalisations by influenza virus type and subtype in the USA, 2010–19: a repeated cross-sectional study

**DOI:** 10.1016/S2666-5247(23)00187-8

**Published:** 2023-09-25

**Authors:** Kelsey M Sumner, Svetlana Masalovich, Alissa O’Halloran, Rachel Holstein, Arthur Reingold, Pam Daily Kirley, Nisha B Alden, Rachel K Herlihy, James Meek, Kimberly Yousey-Hindes, Evan J Anderson, Kyle P Openo, Maya L Monroe, Lauren Leegwater, Justin Henderson, Ruth Lynfield, Melissa McMahon, Chelsea McMullen, Kathy M Angeles, Nancy L Spina, Kerianne Engesser, Nancy M Bennett, Christina B Felsen, Krista Lung, Eli Shiltz, Ann Thomas, H Keipp Talbot, William Schaffner, Ashley Swain, Andrea George, Melissa A Rolfes, Carrie Reed, Shikha Garg

**Affiliations:** Influenza Division (K M Sumner PhD, S Masalovich MS, A O’Halloran MSPH, R Holstein MPH, M A Rolfes PhD, C Reed DSc, S Garg MD), Epidemic Intelligence Service (K M Sumner), US Centers for Disease Control and Prevention, Atlanta, GA, USA; School of Public Health, University of California Berkeley, Berkeley, CA, USA (A Reingold MD); California Emerging Infections Program, Oakland, CA, USA (P D Kirley MPH); Colorado Department of Public Health and Environment, Denver, CA, USA (N B Alden MPH, R K Herlihy MD); Connecticut Emerging Infections Program, Yale School of Public Health, New Haven, CT, USA (J Meek MPH, K Yousey-Hindes MPH); Department of Medicine and Depatment of Pediatrics (E J Anderson MD), Division of Infectious Diseases (K P Openo DrPH), Emory University School of Medicine, Atlanta, GA, USA; Georgia Emerging Infections Program, Georgia Department of Public Health, Atlanta, GA, USA (E J Anderson, K P Openo); Veterans Affairs Medical Center, Atlanta, GA, USA (E J Anderson, K P Openo); Maryland Department of Health, Baltimore, MD, USA (M L Monroe MPH); Michigan Department of Health and Human Services, Lansing, MI, USA (L Leegwater MPH, J Henderson MPH); Minnesota Department of Health, Saint Paul, MN, USA (R Lynfield MD, M McMahon MPH); New Mexico Department of Health, Santa Fe, NM, USA (C McMullen MS); New Mexico Emerging Infections Program, University of New Mexico, Albuquerque, NM, USA (K M Angeles MPH); New York State Department of Health, Albany, NY, USA (N L Spina MPH, K Engesser MPH); University of Rochester School of Medicine and Dentistry, Rochester, NY, USA (N M Bennett MD, C B Felsen MPH); Ohio Department of Health, Columbus, OH, USA (K Lung MPH, E Shiltz MPH); Oregon Health Authority, Salem, OR, USA (A Thomas MD); Vanderbilt University Medical Center, Nashville, TN, USA (H K Talbot MD, W Schaffner MD); Salt Lake County Health Department, Salt Lake City, UT, USA (A Swain CHES, A George MPH)

## Abstract

**Background:**

Influenza burden varies across seasons, partly due to differences in circulating influenza virus types or subtypes. Using data from the US population-based surveillance system, Influenza Hospitalization Surveillance Network (FluSurv-NET), we aimed to assess the severity of influenza-associated outcomes in individuals hospitalised with laboratory-confirmed influenza virus infections during the 2010–11 to 2018–19 influenza seasons.

**Methods:**

To evaluate the association between influenza virus type or subtype causing the infection (influenza A H3N2, A H1N1pdm09, and B viruses) and in-hospital severity outcomes (intensive care unit [ICU] admission, use of mechanical ventilation or extracorporeal membrane oxygenation [ECMO], and death), we used FluSurv-NET to capture data for laboratory-confirmed influenza-associated hospitalisations from the 2010–11 to 2018–19 influenza seasons for individuals of all ages living in select counties in 13 US states. All individuals had to have an influenza virus test within 14 days before or during their hospital stay and an admission date between Oct 1 and April 30 of an influenza season. Exclusion criteria were individuals who did not have a complete chart review; cases from sites that contributed data for three or fewer seasons; hospital-onset cases; cases with unidentified influenza type; cases of multiple influenza virus type or subtype co-infection; or individuals younger than 6 months and ineligible for the influenza vaccine. Logistic regression models adjusted for influenza season, influenza vaccination status, age, and FluSurv-NET site compared odds of in-hospital severity by virus type or subtype. When missing, influenza A subtypes were imputed using chained equations of known subtypes by season.

**Findings:**

Data for 122 941 individuals hospitalised with influenza were captured in FluSurv-NET from the 2010–11 to 2018–19 seasons; after exclusions were applied, 107 941 individuals remained and underwent influenza A virus imputation when missing A subtype (43·4%). After imputation, data for 104 969 remained and were included in the final analytic sample. Averaging across imputed datasets, 57·7% (weighted percentage) had influenza A H3N2, 24·6% had influenza A H1N1pdm09, and 17·7% had influenza B virus infections; 16·7% required ICU admission, 6·5% received mechanical ventilation or ECMO, and 3·0% died (95% CIs had a range of less than 0·1% and are not displayed). Individuals with A H1N1pdm09 had higher odds of in-hospital severe outcomes than those with A H3N2: adjusted odds ratios (ORs) for A H1N1pdm09 versus A H3N2 were 1·42 (95% CI 1·32–1·52) for ICU admission; 1·79 (1·60–2·00) for mechanical ventilation or ECMO use; and 1·25 (1·07–1·46) for death. The adjusted ORs for individuals infected with influenza B versus influenza A H3N2 were 1·06 (95% CI 1·01–1·12) for ICU admission, 1·14 (1·05–1·24) for mechanical ventilation or ECMO use, and 1·18 (1·07–1·31) for death.

**Interpretation:**

Despite a higher burden of hospitalisations with influenza A H3N2, we found an increased likelihood of in-hospital severe outcomes in individuals hospitalised with influenza A H1N1pdm09 or influenza B virus. Thus, it is important for individuals to receive an annual influenza vaccine and for health-care providers to provide early antiviral treatment for patients with suspected influenza who are at increased risk of severe outcomes, not only when there is high influenza A H3N2 virus circulation but also when influenza A H1N1pdm09 and influenza B viruses are circulating.

## Introduction

In the USA, in the decade before the COVID-19 pandemic (2010–20), influenza virus infection was estimated to cause on average up to 710 000 admissions to hospital and 52 000 deaths each year.^1^ The burden of influenza varies greatly from season to season^2^ because of differences in circulating influenza virus types and subtypes as well as differences in influenza vaccine coverage and effectiveness,^3^ with some years seeing lower vaccine effectiveness and higher hospitalisation and mortality rates when influenza A H3N2 viruses are predominant.^4,5^ Despite the effect of virus type or subtype on the yearly influenza burden, little is known about the relative severity of influenza by virus type and subtype in hospitalised individuals. Previous studies^6–12^ have assessed risk factors for severe influenza in hospitalised individuals by virus type; however, many of these studies only focused on adults,^6–8,12^ were set in Europe or Australia,^6–9,11,12^ or assessed only one season;^10^ thus, they are not necessarily representative of influenza severity in children and adults throughout other regions. It is important to understand the severity of influenza infections that lead to hospitalisations across influenza types and subtypes to inform public health messaging and hospital preparation for upcoming influenza seasons.

Using hospitalisation data captured during the 2010–11 to 2018–19 influenza seasons in the US Influenza Hospitalization Surveillance Network (FluSurv-NET), we investigated the relative severity of influenza virus infection by virus type and subtype in both children and adults admitted to hospital with laboratory-confirmed influenza in the USA. Specifically, we described the demographic characteristics and clinical features and complications of individuals hospitalised with laboratory-confirmed influenza according to infecting virus type or subtype and in-hospital severity; and assessed the association between influenza virus type or subtype and in-hospital severity of disease.

## Methods

### Study design

FluSurv-NET is a population-based surveillance system that actively captures laboratory-confirmed, influenza-associated hospitalisations for individuals of all ages in more than 250 acute-care hospitals in the USA. The system represents over 29 million individuals, or approximately 9% of the US population. FluSurv-NET cases are hospitalised residents of the surveillance catchment area with a positive influenza virus test within 14 days before or during their hospital stay and an admission date between Oct 1 and April 30 of an influenza season. Influenza test results are ordered at the treating health-care provider’s discretion. Positive test results can be from a rapid antigen test, molecular testing, viral culture, or direct or indirect fluorescent staining assay. Surveillance staff identify cases by querying infection control and hospital discharge databases within the FluSurv-NET catchment area hospitals as well as state-wide health information exchanges, electronic laboratory reporting databases, and reportable condition databases. Medical record abstraction is performed by trained surveillance staff using a standard case report form to collect demographic information, underlying conditions, interventions, and outcomes identified while individuals are in the hospital.

For this analysis, we captured data for influenza-associated hospitalisations from the 2010–11 to 2018–19 influenza seasons from FluSurv-NET sites in the following states: California, Colorado, Connecticut, Georgia, Maryland, Michigan, Minnesota, Ohio, Oregon, New Mexico, New York, Tennessee, and Utah. Sites that contributed data for three or fewer influenza seasons were excluded from this analysis. For the 2010–11 to 2016–17 influenza seasons, complete medical record abstraction was performed for all identified FluSurv-NET cases. For the 2017–18 and 2018–19 influenza seasons, to reduce the burden on site staff, selected sites had complete medical record abstraction only performed for all individuals younger than 50 years, those who died (at any age) during hospitalisation or within 30 days of discharge, and for a random sample of individuals aged 50 years and older.^13^ Sampling weights were created to adjust analyses by the probability of an individual being selected for complete medical record abstraction, where each individual’s sampling weight was the inverse of the probability of selection for complete abstraction. Additional details on the FluSurv-NET sampling scheme have been previously published.^14^

Additionally, individuals were excluded if they had hospital-onset influenza (defined as a positive influenza test >3 days after hospital admission), did not have a complete chart review because they were not sampled, influenza type could not be identified, were co-infected with multiple influenza virus types or subtypes, or were younger than 6 months at the time of hospitalisation and ineligible for influenza vaccination. Co-infections with other viruses were not assessed so this was not an exclusion criterion.

FluSurv-NET sites obtained participant and ethics approvals from their respective state health department and academic partner Institutional Review Boards (IRBs) as needed. The US Centers for Disease Control and Prevention (CDC) determined this activity met the requirement for public health surveillance; therefore, the CDC’s IRB approval was not required.

### Procedures

The main exposure of interest was influenza virus type or subtype, categorized as A H3N2, A H1N1pdm09, or B. The main outcomes of interest were: (1) admission to an intensive care unit (ICU); (2) receipt of invasive mechanical ventilation or extracorporeal membrane oxygenation (ECMO); and (3) death during hospitalisation.

The following covariates were assessed as potential risk factors associated with severe, influenza-associated, in-hospital outcomes: age (6 months–17 years, 18–49 years, 50–64 years, or ≥65 years), sex, race and ethnicity (White, non-Hispanic; Black, non-Hispanic; Native American or Alaskan Native, non-Hispanic; Asian or Pacific Islander, non-Hispanic; multiracial, non-Hispanic; Hispanic or Latino; or unknown), presence of an underlying medical condition (no comorbid conditions or ≥1 comorbid condition: lung disease, metabolic disease, blood disorder, cardiovascular disease, neuromuscular disorder, neurologic disease, immunocompromised condition, renal disease, liver disease, obesity, and extreme obesity [among those aged ≥2 years]), pneumonia during current hospitalisation, seasonal influenza vaccination status (defined as current season vaccination ≥14 days before a positive influenza test among individuals ≥6 months old), antiviral treatment, and influenza season.

### Statistical analysis

Descriptive tables were created to compare risk factors for severe in-hospital outcomes stratified by influenza virus type or subtype, with counts presented unweighted and percentages weighted by FluSurv-NET’s sampling design. Next, we assessed the adjusted association between influenza virus type or subtype and severe in-hospital outcomes by controlling for confounding variables; potential confounding factors were examined and chosen using directed acyclic graph analysis (appendix p 12). Confounders identified were the individual’s age (categorised as mentioned previously), receipt of the season’s influenza vaccine, influenza season, and FluSurv-NET site.

Because influenza A subtype, seasonal influenza vaccination status, ICU admission, mechanical ventilation or ECMO use, and death had missing observations, we conducted analyses to determine whether multiple imputation was appropriate. We first assessed the pattern of missing data and the mechanism of missingness across influenza A subtype using a χ^2^ test with Rao and Scott’s second-order correction. We also compared covariate distributions among individuals with missing and observed A subtype data and then regressed the indicator of missing A subtype on other variables in the dataset. After multiple imputation, we compared the parameter estimates produced by logistic regression between complete case analysis and the analysis of multiply imputed data. The proportion of unimputed and imputed A subtypes by season was also compared.

We used multiple imputation by chained equations to impute missing data for variables included in the adjusted analysis model with a focus on imputing influenza A subtype.^15^ Race and ethnicity data and presence of underlying medical conditions were also imputed because they were included in the imputation model and had missing observations. The imputation model included all outcome variables (ICU admission, mechanical ventilation or ECMO, and death), confounding covariates from the analysis models (individual’s age, categorised into finer groups: 0–4 years, 5–17 years, 18–49 years, 50–64 years, 65–74 years, 75–84 years, and ≥85 years, seasonal influenza vaccination status, influenza season, and FluSurv-NET site), and other variables associated with A subtype missingness (sex, race and ethnicity, presence of an underlying medical condition, influenza antiviral use, pneumonia presence, and month of hospital admission [grouped as October to December, January, February, and March to April]), and the sampling weight for the individual. These additional variables were selected based on their correlation with other variables in the dataset (ie, tetrachoric and polychoric correlation coefficients ≥0·3) and their association with indicators for missingness (ie, assessed as statistically significant using logistic regression). Covariates in the imputation model with missing observations were imputed sequentially using logistic regression, with the covariate with the least amount of missing data imputed first. 30 imputed datasets were created. After imputation, we derived a three-category version of the influenza type or subtype variable encompassing influenza A H3N2, A H1N1pdm09, and B viruses. Influenza B lineage was not captured by FluSurv-NET and thus not imputed.

After imputation, to investigate the adjusted odds ratio (OR) between influenza type or subtype and severe in-hospital outcomes, we ran logistic regression models accounting for the complex survey design and adjusted for influenza season, seasonal influenza vaccination status, individual’s age, and FluSurv-NET site on each imputed dataset; parameter estimates were summarised using Rubin’s rule.^16^ Each model was weighted using sampling weights created from sampling strata on the basis of whether an individual died, the individual’s age, the influenza season, and FluSurv-NET site. The adjusted association was assessed for effect measure modification on the multiplicative scale by individual’s age, influenza season, and seasonal influenza vaccination status; to do so, we compared adjusted ORs and 95% CIs stratified by age, season, or seasonal influenza vaccination status with the full model adjusted OR.

Sensitivity analyses were conducted to evaluate whether having a pre-existing medical condition or receipt of influenza antiviral drugs changed results, with an additional covariate for each added to the adjusted logistic regression models. Models that were additionally adjusted for having a pre-existing medical condition were also assessed stratified by age. To assess the performance of the imputation model, we performed complete case analysis of the adjusted association between influenza type or subtype with severe in-hospital outcomes and compared results with effect estimates produced using the imputed datasets. Data analyses were performed using SAS version 9.4 software (SAS Institute, Cary, NC, USA) and the ‘mice’ and ‘survey’ packages in R version 4.0.3 software (R Core Team, Vienna, Austria).^15,17^ For all statistical analyses, statistical significance was assessed at an α level of 0·05. STROBE and RECORD reporting guidelines were followed in the generation of this report.

### Role of the funding source

The funder of the study had a role in study design, data collection, data analysis, data interpretation, and writing of the report.

## Results

**Figure 1:**
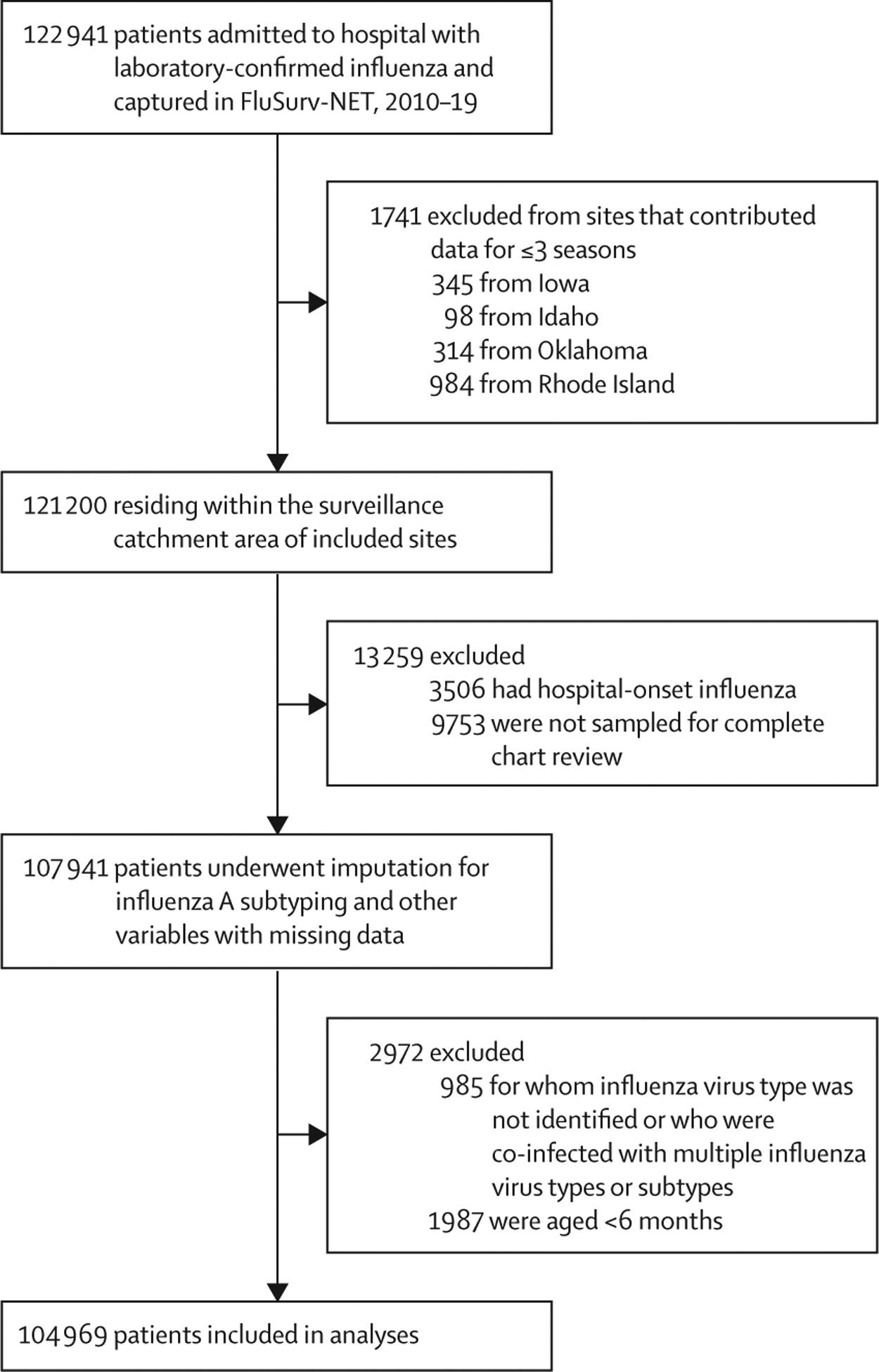
Study profile Data are for individuals hospitalised with laboratory-confirmed influenza captured in the US Influenza Hospitalization Surveillance Network, 2010–19. Main analysis model covariates that were imputed were influenza A virus subtype, seasonal influenza vaccination status, mechanical ventilation or use of extracorporeal membrane oxygenation, admission to an intensive care unit, and death. Race and ethnicity, and presence of at least one underlying medical condition, were part of the imputation model and imputed due to missingness.

Data for 122 941 hospitalised individuals with laboratory-confirmed influenza virus infection were captured in FluSurv-NET from the 2010–11 to 2018–19 seasons (figure 1). After exclusions were applied, 107 941 individuals remained in the dataset (figure 1); about half of these individuals were female and most had at least one comorbid condition (table 1). 25·9% (weighted percentage) of individuals were hospitalised with influenza A H3N2, 11·9% with influenza A H1N1pdm09, 43·4% with influenza A unknown subtype, 18·2% with influenza B, 0·4% with influenza A and B, and 0·3% with influenza of unknown virus type (table 1). Specific comorbid conditions across influenza virus type or subtype are described in the appendix (p 2). ICU admission occurred in 16·2% (weighted percentage), mechanical ventilation or ECMO use in 6·2%, and death in 2·7% of 107 941 hospitalisations.

**Figure 2:**
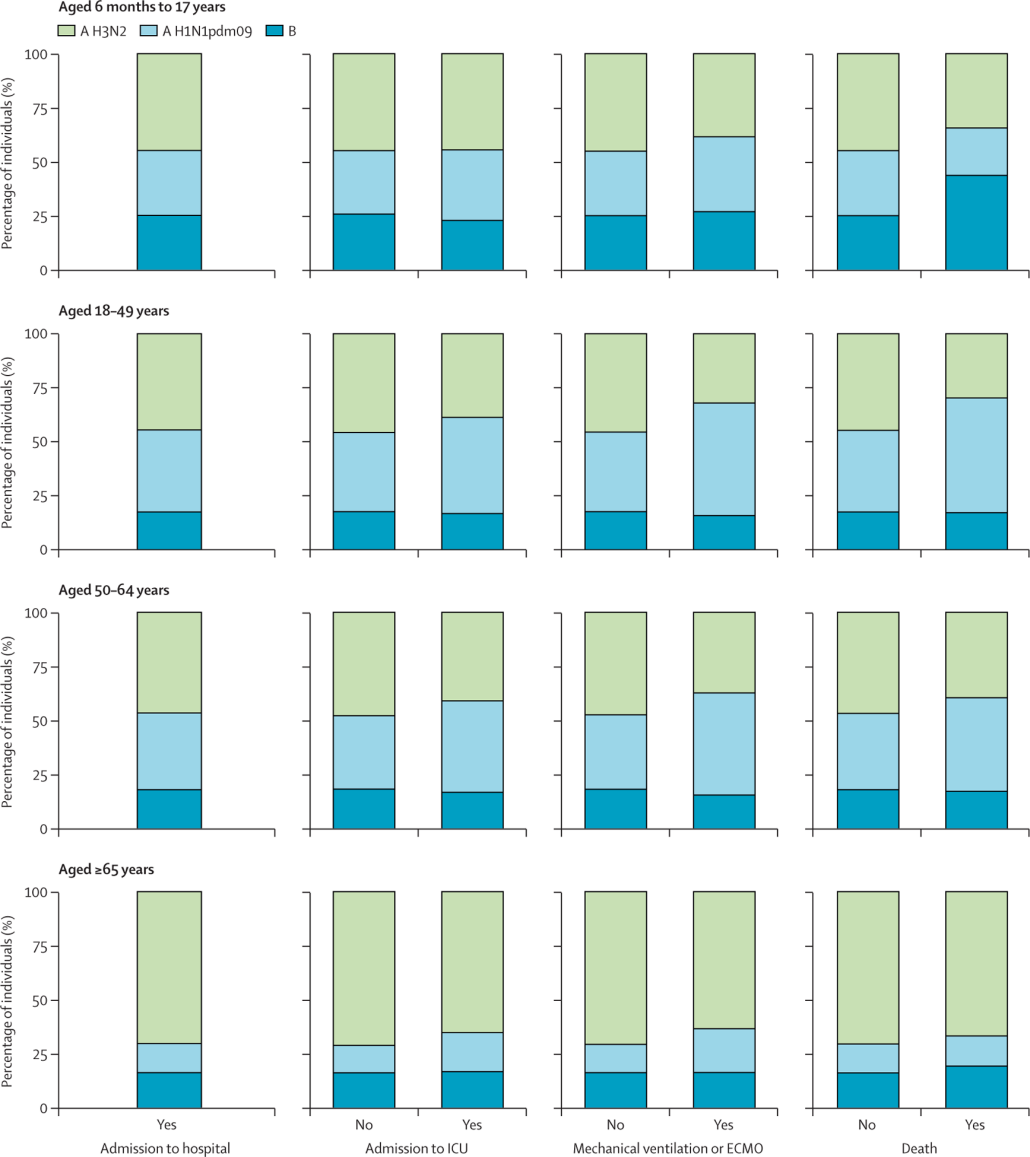
Distribution of imputed influenza A virus subtypes and influenza B virus infections, stratified by age of the individual and in-hospital outcome Data are for all captured hospitalisations, those admitted to an ICU, those who received mechanical ventilation or ECMO, and those who died in the hospital. Across each age group, the mean proportion of hospitalised individuals with influenza A H3N2, A H1N1pdm09, and influenza B virus infections is shown across all hospitalisations and across the presence or absence of each severity measure. The mean percentages of influenza types and imputed subtypes were calculated across the 30 imputed data sets; 95% CIs had a range of less than 0·1% and are not displayed in the figures. ICU=intensive care unit. ECMO=extracorporeal membrane oxygenation.

Influenza A subtype was imputed for 43·4% (weighted percentage) of hospitalisations. The pattern of influenza A subtype missingness was non-monotone and probably not missing completely at random (appendix p 4). After influenza A subtype imputation, data for 104 969 remained and were included in the final analytic sample (figure 1). After imputation, averaging across imputed datasets, 57·7% (weighted percentage) of hospitalised individuals had A H3N2, 24·6% had A H1N1pdm09, and 17·7% had influenza B infections; 16·7% required ICU admission, 6·5% received mechanical ventilation or ECMO, and 3·0% died (95% CIs had a range of less than 0·1% and are not displayed). Distributions of imputed influenza virus type or subtype varied by outcome and age group (figure 2).

Using the imputed datasets, and adjusting for influenza season, seasonal influenza vaccination status, the individual’s age, and FluSurv-NET site, we found that individuals with influenza A H1N1pdm09 virus infections had higher odds of in-hospital severe outcomes of ICU admission, mechanical ventilation or ECMO, and death than those with A H3N2 virus infection (table 2, appendix p 6). Individuals infected with influenza B virus also had higher odds of in-hospital severe outcomes than those with influenza A H3N2 virus (table 2, appendix p 6).

Comparing the association of influenza B virus versus A H3N2 virus and severe in-hospital outcomes across age groups, higher odds of mechanical ventilation or ECMO use were observed in children aged 6 months–17 years and adults aged 18–49 years (table 2). Higher odds of death comparing individuals hospitalised with influenza B versus A H3N2 virus were observed in children aged 6 months to 17 years and adults aged 65 years and older (table 2). By contrast, no association was found comparing odds of death in individuals aged 6 months to 17 years and aged 65 years and older hospitalised with A H1N1pdm09 versus A H3N2 virus; however, the adjusted OR was significant for death in individuals aged 18–49 and 50–64 years.

In analyses stratified by influenza vaccination status, individuals who did not receive the seasonal influenza vaccine had higher odds of death when infected with influenza A H1N1pdm09 or B virus compared with A H3N2 virus (table 2). Associations were less strong for those who had received the seasonal influenza vaccine (table 2). Occasionally, variation in the association of influenza type or subtype with severe in-hospital outcomes was observed across influenza seasons (table 2).

In sensitivity analyses, when adjusting for the presence of at least one underlying medical condition, model results were similar for the outcomes of ICU admission, mechanical ventilation or ECMO use, and death (appendix p 8) even after stratifying by age (appendix p 9). Adjusting for antiviral use also produced results in the same direction of effect across all outcomes, including ICU admission, mechanical ventilation or ECMO use, and death (appendix p 8).

In a complete case sensitivity analysis using the original, unimputed dataset, increased odds of severe in-hospital outcomes were observed when comparing individuals with influenza A H1N1pdm09 with those with A H3N2 virus (adjusted ORs: ICU admission 1·40, 95% CI 1·29–1·51; mechanical ventilation or ECMO use 1·77, 1·58–1·99; death 1·30, 1·10–1·54) but not when comparing influenza B with A H3N2 virus (adjusted ORs: ICU admission 0·95, 95% CI 0·89–1·01; mechanical ventilation or ECMO use 0·99, 0·90–1·09; death 1·14, 1·00–1·30; appendix pp 10–11).

## Discussion

Across nearly 105 000 influenza hospitalisations and nine influenza seasons from 2010 to 2019 in the USA, we found that more than one in six individuals hospitalised with influenza had severe outcomes. Although influenza A H3N2 virus-predominant seasons are typically associated with more hospitalisations overall, we found that in-hospital severity was greater among individuals infected with influenza A H1N1pdm09 virus or influenza B virus. Higher in-hospital severity in individuals with A H1N1pdm09 occurred across all age groups when severity was measured as ICU admission, or mechanical ventilation or ECMO use, although results were variable for B and when severity was measured as in-hospital death.

Previous studies noted more severe influenza illness when adults^7,8,12^ or both children and adults^9–11,18,19^ were hospitalised with influenza A H1N1pdm09 virus compared with A H3N2 virus; however, some of these studies found that results varied depending on the severity outcome measured^7,18^ or age of participants.^8^ We observed similar variability across age and outcome measures, where all ages had increased odds of ICU admission and mechanical ventilation or ECMO use with A H1N1pdm09 virus versus A H3N2 virus, but neither young children nor older adults had increased odds of death; this difference in the association between virus type or subtype and death across age groups could be partly explained by immune imprinting of the specific influenza viruses that individuals were first exposed to in childhood.^20,21^ An evaluation of the effect of immune imprinting on influenza severity was outside the scope of this study but could be investigated in future work. The increased odds of ICU admission and mechanical ventilation or ECMO use that we noted across all ages hospitalised with influenza A H1N1pdm09 virus infections compared with influenza A H3N2 virus infections could be related to the increased infiltration of pneumocytes and intra-alevolar macrophages with influenza A H1N1pdm09 virus, causing alveoli inflammation and more severe disease manifestation, as has been noted in animal models.^22,23^ Future studies could further investigate the biological mechanisms for increased severity of influenza A H1N1pdm09 compared with A H3N2 virus in hospitalised individuals with influenza.

We noted that children younger than 18 years and adults aged 65 years and older infected with influenza B viruses had higher odds of death compared with those infected with A H3N2. Increased odds of death in children with influenza B have been detected before; during the 2010–11 to 2017–18 US influenza seasons, a review^24^ showed that the proportion of paediatric deaths in individuals infected with influenza B virus was disproportionately higher than the fraction of circulating viruses that were influenza B. In Greece, older adults have also accounted for a disproportionately high number of deaths from influenza B virus infections, as noted over nine seasons.^11^ Other previous studies found no association with disease severity between infections with influenza B virus versus influenza A^25,26^ or A H3N2 viruses;^10^ however, these studies did not present comparisons of the influenza type–severity relationship stratified by age groups.^10,25,26^ We concluded that the relative severity between influenza B and A H3N2 virus infections might be heterogeneous by age, as our age-stratified estimates were statistically significant only for selected age groups and severity measures.

Typically, lower vaccine effectiveness and subsequently higher hospitalisation rates are associated with influenza A H3N2-predominant seasons.^4^ Although we noted a higher number of hospitalisations from infections with influenza A H3N2 virus in our study, we found that once individuals were hospitalised, those with influenza A H1N1pdm09 and B virus infections were more likely to have severe outcomes. Future work could evaluate how the likelihood of hospitalisation compared with in-hospital outcomes differs by influenza type or subtype. Although the reasons for increased odds of severe illness when infected with A H1N1pdm09 or B virus are unclear, it is important for individuals to take precautions to protect themselves against influenza virus infection not only when A H3N2 virus is predominant, but also when A H1N1pdm09 and B viruses are circulating. During the influenza season, increased subtyping to aid early identification of the influenza virus type or subtype infecting most hospitalised individuals could inform planning for hospitals as well as public health messaging aimed at health-care providers around vaccination and appropriate antiviral treatment.^10,27^ Finally, we noted higher odds of death in unvaccinated individuals who were hospitalised with influenza A H1N1pdm09 or B compared with A H3N2 virus. No association between influenza type or subtype and odds of death was noted in vaccinated individuals, highlighting the importance of receiving the annual influenza vaccine to protect against severe illness.

This study had some limitations. FluSurv-NET, which relies on clinical testing for influenza, probably under-ascertains influenza-associated hospitalisations, as we know that not all individuals hospitalised with acute respiratory illness during the influenza season are tested for influenza.^28^ Any case under-ascertainment that might have occurred was probably irrespective of influenza type or subtype and we do not believe it affected results. Co-infection with other viruses was not assessed and could have affected an individual’s likelihood of having a severe in-hospital outcome. Influenza A subtype information was missing for more than 40% of hospitalised individuals and required imputation, which could have misclassified influenza A subtypes; however, sensitivity analyses found similar model results comparing influenza A H1N1pdm09 with A(H3N2) proportions by season, indicating that misclassification was probably low. Previous studies have successfully imputed the high missingness of influenza A subtype in FluSurv-NET.^13,14,29^ Additionally, differences in vaccine effectiveness by season could have varied by type or subtype. Although we were able to adjust for influenza vaccination and season, we were unable to adjust estimates for annual vaccine effectiveness. Vaccine effectiveness is typically lower for influenza A H3N2 than for A H1N1pdm09 virus,^30^ which, when not accounted for, would increase the likelihood of individuals having more severe A H3N2 virus infections and bias our results towards the null. Influenza B lineage was not captured by FluSurv-NET, so differences in influenza severity by B lineage could not be assessed. Finally, we were unable to assess the relative risk of hospitalisation with A H3N2 virus compared with A H1N1pdm09 or B because we did not have population level data on prevalence of infection by influenza type or subtype. Future analyses could assess the relative risk of hospitalisation by influenza subtype.

Overall, differences in in-hospital disease severity were noted by influenza type and subtype, with higher odds of severe disease in individuals hospitalised with influenza A H1N1pdm09 and B viruses than in those with influenza A H3N2 virus. Thus, it is important for individuals to receive an annual influenza vaccine and for health-care providers to prescribe early antiviral treatment for patients with suspected influenza who are at higher risk for complications, not only when there is high influenza A H3N2 virus circulation but also when influenza A H1N1pdm09 and B viruses are circulating. Early season detection of influenza type and subtype can inform public health messaging and vaccination campaigns to reduce influenza-associated hospitalisations and severe in-hospital outcomes; this is especially important now with co-circulation of other respiratory viruses, such as SARS-CoV-2 and respiratory syncytial virus, adding to the respiratory disease burden in hospital settings.

## Supplementary Material

1

## Figures and Tables

**Table 1:** Characteristics of individuals hospitalised with laboratory-confirmed influenza in the USA, 2010–19, by infecting virus type and unimputed subtype*

	Total cases (n=107 941)	Influenza A (n=88 276)			Influenza B (n=18 909)	Influenza A and B (n=441)	Unknown type (n=315)
		H3N2 (n=28 409)	H1N1pdm09 (n=13 597)	Unknown A subtype (n=46 270)			
**Demographic characteristics**							
Age, years							
0–17	13 236 (11·2%)	3080 (10·1%)	2121 (15·1%)	4749 (9·3%)	3090 (14·5%)	75 (15·7%)	121 (37·5%)
18–49	19 438 (16·5%)	3869 (12·7%)	3752 (26·7%)	8327 (16·3%)	3360 (15·7%)	90 (18·8%)	40 (12·4%)
50–64	23 422 (21·0%)	5013 (17·1%)	4295 (31·3%)	9833 (20·4%)	4139 (21·5%)	95 (21·7%)	47 (14·8%)
≥65	51 845 (51·2%)	16 447 (60·1%)	3429 (26·9%)	23 361 (54·0%)	8320 (48·3%)	181 (43·7%)	107 (35·3%)
Sex							
Male	49 814 (46·1%)	12 977 (45·6%)	6626 (48·7%)	21 132 (45·7%)	8720 (46·0%)	198 (43·6%)	161 (51·1%)
Female	58 127 (53·9%)	15 432 (54·4%)	6971 (51·3%)	25 138 (54·3%)	10 189 (54·0%)	243 (56·4%)	154 (48·9%)
Race or ethnicity							
White, non-Hispanic	61 951 (58·1%)	17 179 (61·4%)	7360 (54·4%)	26 563 (58·0%)	10 451 (56·2%)	243 (52·7%)	155 (49·5%)
Black, non-Hispanic	21 682 (19·7%)	4936 (16·9%)	2972 (21·7%)	9533 (20·2%)	4067 (20·8%)	103 (23·9%)	71 (22·0%)
Native American or Alaskan Native, non-Hispanic	718 (0·7%)	137 (0·5%)	118 (0·9%)	362 (0·8%)	90 (0·4%)	8 (1·7%)	3 (0·9%)
Asian or Pacific Islander, non-Hispanic	4808 (4·8%)	1155 (4·1%)	544 (4·1%)	2239 (5·3%)	833 (5·1%)	23 (5·8%)	14 (4·3%)
Multiracial, non-Hispanic	391 (0·3%)	86 (0·3%)	53 (0·4%)	174 (0·4%)	75 (0·4%)	2 (0·4%)	1 (0·3%)
Hispanic or Latino	9753 (8·9%)	2232 (7·8%)	1548 (11·2%)	4103 (8·7%)	1789 (9·3%)	41 (11·0%)	40 (13·3%)
Unknown	8638 (7·6%)	2684 (9·1%)	1002 (7·3%)	3296 (6·7%)	1604 (7·9%)	21 (4·4%)	31 (9·6%)
Influenza season							
2010–11	5442 (4·6%)	1653 (5·4%)	935 (6·7%)	1871 (3·7%)	917 (4·3%)	27 (5·7%)	39 (12·1%)
2011–12	2294 (1·9%)	950 (3·1%)	312 (2·2%)	755 (1·5%)	260 (1·2%)	8 (1·7%)	9 (2·8%)
2012–13	11 088 (9·4%)	3816 (12·5%)	165 (1·2%)	4725 (9·3%)	2311 (10·8%)	28 (5·9%)	43 (13·3%)
2013–14	9255 (7·9%)	456 (1·5%)	4460 (31·8%)	3250 (6·4%)	1011 (4·7%)	34 (7·1%)	44 (13·6%)
2014–15	17 235 (14·6%)	6805 (22·3%)	16 (0·1%)	7919 (15·5%)	2356 (11·0%)	103 (21·6%)	36 (11·2%)
2015–16	8506 (7·2%)	427 (1·4%)	2952 (21·0%)	2924 (5·7%)	2129 (10·0%)	51 (10·7%)	23 (7·1%)
2016–17	16 992 (14·4%)	5964 (19·6%)	117 (0·8%)	7199 (14·1%)	3604 (16·9%)	63 (13·2%)	45 (13·9%)
2017–18	20 996 (24·6%)	5269 (22·9%)	1078 (9·2%)	8837 (24·4%)	5685 (37·7%)	88 (25·6%)	39 (13·2%)
2018–19	16 133 (15·3%)	3069 (11·2%)	3562 (27·0%)	8790 (19·5%)	636 (3·4%)	39 (8·6%)	37 (12·7%)
Underlying medical condition							
No comorbid conditions	13 901 (12·3%)	3039 (10·3%)	2166 (15·6%)	5644 (11·6%)	2905 (14·5%)	69 (14·5%)	78 (24·2%)
≥1 comorbid conditions	93 751 (87·4%)	25 297 (89·5%)	11 417 (84·3%)	40 475 (88·0%)	15 954 (85·2%)	372 (85·5%)	236 (75·2%)
Unknown	289 (0·3%)	73 (0·2%)	14 (0·1%)	151 (0·4%)	50 (0·3%)	0 (0·0%)	1 (0·6%)
Seasonal influenza vaccination*							
No	44 087 (40·6%)	10 219 (35·8%)	7273 (54·2%)	18 363 (39·5%)	7911 (41·2%)	185 (42·4%)	136 (44·8%)
Yes	48 212 (46·3%)	14 208 (51·6%)	4561 (34·8%)	21 155 (47·2%)	7981 (44·0%)	173 (41·2%)	134 (45·7%)
Unknown	13613 (13·1%)	3443 (12·5%)	1458 (11·1%)	5921 (13·3%)	2699 (14·8%)	64 (16·4%)	28 (9·5%)
**Influenza complications and interventions**							
Pneumonia during current hospitalisation							
No	84 122 (77·9%)	22 810 (80·3%)	9766 (72·0%)	36 151 (78·0%)	14 842 (78·5%)	317 (72·5%)	236 (74·9%)
Yes	23 819 (22·1%)	5599 (19·7%)	3831 (28·0%)	10 119 (22·0%)	4067 (21·5%)	124 (27·5%)	79 (25·1%)
Antiviral treatment							
No	15 331 (13·5%)	4811 (16·2%)	2171 (15·6%)	4908 (10·1%)	3306 (16·4%)	66 (14·1%)	69 (21·7%)
Yes	92 610 (86·5%)	23 598 (83·8%)	11426 (84·4%)	41 362 (89·9%)	15 603 (83·6%)	375 (85·9%)	246 (78·3%)
**Influenza disease severity**							
ICU admission							
No	89 381 (83·2%)	23 778 (84·1%)	10 425 (76·8%)	38 868 (84·4%)	15 702 (83·5%)	341 (77·9%)	267 (84·5%)
Yes	17 971 (16·2%)	4466 (15·3%)	3131 (22·9%)	7133 (15·0%)	3097 (15·9%)	97 (21·4%)	47 (14·9%)
Unknown	589 (0·6%)	165 (0·5%)	41 (0·3%)	269 (0·6%)	110 (0·6%)	3 (0·6%)	1 (0·6%)
Mechanical ventilation or ECMO							
No	10 0228 (93·1%)	26 571 (93·7%)	12 068 (88·8%)	43 275 (93·7%)	17 620 (93·4%)	397 (90·8%)	297 (93·8%)
Yes	6968 (6·2%)	1613 (5·6%)	1467 (10·8%)	2669 (5·5%)	1161 (5·9%)	41 (8·6%)	17 (5·6%)
Unknown	745 (0·7%)	225 (0·8%)	62 (0·5%)	326 (0·8%)	128 (0·7%)	3 (0·6%)	1 (0·6%)
Death, n (%)							
No	10 4360 (96·9%)	27 445 (96·8%)	13166 (96·9%)	44 783 (97·1%)	18 248 (96·9%)	412 (93·9%)	306 (96·9%)
Yes	3227 (2·7%)	859 (2·8%)	405 (2·9%)	1323 (2·6%)	606 (2·8%)	26 (5·4%)	8 (2·5%)
Unknown	354 (0·3%)	105 (0·3%)	26 (0·2%)	164 (0·3%)	55 (0·3%)	3 (0·6%)	1 (0·6%)
Days hospitalised, median (IQR)	3 (3)	3 (3)	3 (4)	3 (3)	3 (3)	3 (4)	3 (3)

Data are n (%) unless otherwise stated. Counts are presented unweighted, whereas percentages were weighted using complex survey weights. Median and IQR presented were weighted using complex survey weights. Data presented are before imputation. ICU=intensive care unit. ECMO=extracorporeal membrane oxygenation.

* Only captured for individuals aged 6 months or older; 2029 observations from infants younger than 6 months were excluded.

**Table 2:** Adjusted odds ratios of severe outcomes by influenza type and imputed influenza A virus subtype for individuals hospitalised with laboratory-confirmed influenza in the USA, 2010–19

	ICU admission		Mechanical ventilation or ECMO use		Death	
	A H1N1pdm09 *vs* A H3N2	B *vs* A H3N2	A H1N1pdm09 *vs* A H3N2	B *vs* A H3N2	A H1N1pdm09 *vs* A H3N2	B *vs* A H3N2
Overall	1·42 (1·32–1·52)	1·06 (1·01–1·12)	1·79 (1·60–2·00)	1·14 (1·05–1·24)	1·25 (1·07–1·46)	1·18 (1·07–1·31)
Age						
6 months to 17 years	1·26 (1·08–1·47)	0·92 (0·82–1·04)	1·67 (1·26–2·22)	1·30 (1·04–1·61)	1·31 (0·54–3·16)	2·55 (1·42–4·57)
18–49 years	1·43 (1·26–1·63)	1·10 (0·98–1·23)	1·79 (1·47–2·18)	1·24 (1·04–1·48)	1·81 (1·14–2·87)	1·39 (0·93–2·06)
50–64 years	1·43 (1·25–1·64)	1·05 (0·95–1·17)	1·77 (1·47–2·14)	1·08 (0·93–1·26)	1·49 (1·13–1·98)	1·11 (0·88–1·41)
≥65 years	1·49 (1·32–1·67)	1·12 (1·03–1·21)	1·83 (1·52–2·20)	1·10 (0·97–1·25)	1·05 (0·87–1·27)	1·15 (1·03–1·30)
Seasonal influenza vaccination status						
No	1·41 (1·29–1·54)	1·06 (0·98–1·14)	1·86 (1·61–2·14)	1·17 (1·05–1·32)	1·49 (1·19–1·87)	1·27 (1·07–1·51)
Yes	1·42 (1·28–1·58)	1·07 (0·99–1·16)	1·68 (1·44–1·95)	1·12 (0·99–1·26)	1·06 (0·87–1·31)	1·12 (0·98–1·29)
Influenza season						
2010–11	1·73 (1·41–2·11)	0·94 (0·74–1·20)	2·01 (1·50–2·68)	1·10 (0·78–1·55)	1·28 (0·81–2·03)	1·20 (0·72–2·01)
2011–12	1·19 (0·83–1·72)	1·29 (0·87–1·90)	1·44 (0·82–2·52)	1·70 (0·94–3·07)	1·24 (0·49–3·12)	2·08 (0·88–4·91)
2012–13	1·35 (0·97–1·87)	1·03 (0·90–1·18)	1·44 (0·90–2·32)	1·10 (0·90–1·34)	0·90 (0·25–3·27)	1·21 (0·88–1·68)
2013–14	1·39 (1·09–1·77)	0·94 (0·70–1·25)	2·06 (1·38–3·08)	1·36 (0·86–2·13)	0·93 (0·55–1·60)	0·71 (0·38–1·32)
2014–15*	1·29 (0·39–4·24)	1·09 (0·96–1·23)	1·79 (0·44–7·19)	1·13 (0·94–1·35)	0·001 (0·00–348·24)	1·07 (0·81–1·41)
2015–16	1·27 (0·98–1·64)	0·97 (0·74–1·26)	1·71 (1·17–2·48)	1·04 (0·70–1·55)	1·25 (0·70–2·22)	1·13 (0·63–2·04)
2016–17	1·60 (1·13–2·27)	1·00 (0·90–1·11)	1·53 (0·87–2·70)	0·99 (0·84–1·18)	0·86 (0·27–2·72)	1·17 (0·93–1·47)
2017–18	1·46 (1·25–1·71)	1·14 (1·02–1·26)	1·81 (1·39–2·35)	1·25 (1·06–1·48)	1·34 (0·95–1·90)	1·24 (1·05–1·47)
2018–19	1·35 (1·20–1·51)	1·19 (0·94–1·51)	1·69 (1·40–2·04)	1·02 (0·68–1·56)	1·25 (0·98–1·59)	1·24 (0·77–1·99)

Data are adjusted odds ratios (95% CI), stratified by age, receipt of seasonal influenza vaccine, and influenza season. Odds ratios and corresponding 95% CIs were estimated using logistic regression models accounting for survey weights and imputation for each outcome separately. Results were pooled across 30 imputed datasets using Rubin’s rules. Each model included influenza type and subtype, age (6 months to 17 years, 18–49 years, 50–64 years, and ≥65 years), seasonal influenza vaccination status, influenza season, and FluSurv-NET site. ICU=intensive care unit. ECMO=extracorporeal membrane oxygenation.

* Very few influenza A H1N1pdm09 viruses circulated during the 2014–15 influenza season.

## Data Availability

Individuals interested in receiving a limited dataset can submit a brief proposal to the corresponding author (KMS) for review and consideration by the CDC and FluSurv-NET site partners.

## Research in context

### Evidence before this study

Influenza virus type or subtype circulation and hospitalisation burden varies by season, but information is scarce regarding how severe influenza outcomes, and thus use of intensive care unit resources, differ by influenza type or subtype once individuals are admitted to hospital. We did a PubMed search of “influenza” AND “severity” AND (“type” OR “subtype”) of studies published from database inception to Dec 31, 2022, with no language restrictions. 2066 articles met the PubMed search criteria; eight of these were research articles that investigated severe influenza in hospitalised individuals by virus type. These articles were predominantly set in Europe and Australia. The previous studies mainly focused on adults, smaller populations, or few seasons.

### Added value of this study

We built on the previous studies by assessing the association between influenza virus type or subtype and in-hospital severity for nearly 105 000 patients of all ages with influenza admitted to hospital across nine influenza seasons in the USA.

### Implications of all the available evidence

We showed that, although influenza A H3N2 virus-predominant seasons are typically associated with more hospitalisations overall, in-hospital severity was greater in individuals infected with influenza A H1N1pdm09 or influenza B viruses compared with influenza A H3N2 virus. Thus, it is important for individuals to receive an annual influenza vaccine and for health-care providers to provide early antiviral treatment for high-risk patients with suspected influenza, not only when there is high circulation of A H3N2 virus, but also when A H1N1pdm09 and B viruses are circulating.
