# Supplementary material for: Severity of influenza-associated hospitalisations by influenza virus type and subtype in the USA, 2010–19: a repeated cross-sectional study
Source: Lancet Microbe. Author manuscript; Available in PMC 2024 Feb 16. (PMC10872935; doi:10.1016/S2666-5247(23)00187-8)

THE LANCET
Microbe

Supplementary appendix

This appendix formed part of the original submission and has been peer reviewed. We post it as supplied by the authors.

Supplement to: Sumner KM, Masalovich S, O'Halloran A, et al. Severity of influenza-associated hospitalisations by influenza virus type and subtype in the USA, 2010–19: a repeated cross-sectional study. *Lancet Microbe* 2023; published online Sept 25. https://doi.org/10.1016/S2666-5247(23)00187-8.

APPENDIX

Table of contents

Supplemental tables 2

Supplemental table 1. Characteristics of specific comorbid conditions among individuals hospitalized with laboratory-confirmed influenza by infecting virus type and unimputed subtype[a] 2

Supplemental table 2. Characteristics of hospitalized individuals with influenza A by known versus unknown influenza A subtype (before imputation) 4

Supplemental table 3. Full model of adjusted association between influenza type and imputed subtype and odds of severe in-hospital outcomes 6

Supplemental table 4. Adjusted association between influenza type and imputed subtype and odds of severe in-hospital outcomes adding additional covariates to the multivariable model 8

Supplemental table 5. Adjusted odds ratios of severe in-hospital outcomes by influenza type and imputed subtype stratified by age and additionally adjusted for underlying medical conditions 9

Supplemental table 6. Adjusted association between influenza type and subtype and odds of severe in-hospital outcomes using original, unimputed data set that excluded observations missing influenza A subtype 10

Supplemental table 7. Comparison of influenza A subtype proportions before and after imputation by influenza season 11

Supplemental figure 12

Supplemental tables

Supplemental table 1. Characteristics of specific comorbid conditions among individuals hospitalized with laboratory-confirmed influenza by infecting virus type and unimputed subtype[a]

Characteristics	Total cases[b]	Influenza A			Influenza B	Influenza A and B	Unknown type
		H3N2	H1N1pdm09	Unknown A subtype			
	N=107941	N=28409	N=13597	N=46270	N=18909	N=441	N=315
Lung disease, n (%)							
No	79837 (73·5)	20692 (72·5)	10028 (73·2)	34316 (73·5)	14223 (74·7)	313 (70·2)	265 (83·6)
Yes	28104 (26·5)	7717 (27·5)	3569 (26·8)	11954 (26·5)	4686 (25·3)	128 (29·8)	50 (16·4)
Metabolic disease, n (%)							
No	67844 (62·0)	17158 (59·7)	9188 (67·0)	28669 (60·9)	12322 (64·3)	277 (60·8)	230 (71·9)
Yes	40097 (38·0)	11251 (40·3)	4409 (33·0)	17601 (39·1)	6587 (35·7)	164 (39·2)	85 (28·1)
Blood disorder, n (%)							
No	103139 (95·8)	27247 (96·1)	12912 (95·1)	44378 (96·2)	17879 (95·0)	416 (94·8)	307 (97·5)
Yes	4802 (4·2)	1162 (3·9)	685 (4·9)	1892 (3·8)	1030 (5·0)	25 (5·2)	8 (2·5)
Cardiovascular disease, n (%)							
No	63565 (57·4)	15618 (53·9)	9269 (67·4)	26604 (56·0)	11579 (59·0)	256 (56·3)	239 (75·6)
Yes	44376 (42·6)	12791 (46·1)	4328 (32·6)	19666 (44·0)	7330 (41·0)	185 (43·7)	76 (24·4)
Neuromuscular disorder, n (%)							
No	102155 (94·7)	26903 (94·8)	13012 (95·7)	43716 (94·5)	17801 (94·3)	414 (94·3)	309 (98·1)
Yes	5786 (5·3)	1506 (5·2)	585 (4·3)	2554 (5·5)	1108 (5·7)	27 (5·7)	6 (1·9)
Neurologic disease, n (%)[c]							
No	82385 (80·1)	21148 (78·7)	10783 (85·1)	35342 (79·5)	14550 (80·6)	330 (80·5)	232 (84·1)
Yes	20114 (19·9)	5608 (21·3)	1879 (14·9)	9057 (20·5)	3442 (19·4)	84 (19·5)	44 (15·9)
Immunocompromised condition, n (%)							
No	90722 (84·1)	24067 (84·7)	11267 (82·7)	39152 (84·8)	15599 (82·5)	359 (80·9)	278 (88·3)
Yes	17219 (15·9)	4342 (15·3)	2330 (17·3)	7118 (15·2)	3310 (17·5)	82 (19·1)	37 (11·7)
Renal disease, n (%)							
No	89334 (82·0)	23171 (81·1)	11804 (86·4)	37908 (81·2)	15791 (82·4)	387 (86·4)	273 (86·7)
Yes	18607 (18·0)	5238 (18·9)	1793 (13·6)	8362 (18·8)	3118 (17·6)	54 (13·6)	42 (13·3)
Liver disease, n (%)[c]							
No	98612 (96·2)	25848 (96·6)	12084 (95·3)	42694 (96·1)	17316 (96·2)	397 (96·0)	273 (98·9)
Yes	3887 (3·8)	908 (3·4)	578 (4·7)	1705 (3·9)	676 (3·8)	17 (4·0)	3 (1·1)
Obesity, n (%)[d]							
No	67514 (65·7)	18430 (67·8)	7566 (59·4)	28941 (65·3)	12124 (67·7)	274 (66·8)	179 (65·2)
Yes	34905 (33·8)	8683 (31·6)	5112 (40·2)	15136 (34·3)	5755 (31·9)	129 (33·0)	90 (33·7)
Unknown	452 (0·5)	150 (0·5)	50 (0·4)	184 (0·5)	65 (0·4)	1 (0·2)	2 (1·1)
Morbid obesity, n (%)[d]							
No	90489 (88·0)	24319 (89·2)	10745 (84·6)	38838 (87·8)	15986 (88·9)	357 (87·5)	244 (89·2)
Yes	9358 (8·9)	2188 (7·9)	1605 (12·5)	4028 (8·9)	1478 (8·1)	39 (10·1)	20 (7·9)
Unknown	3024 (3·1)	756 (2·9)	378 (2·9)	1395 (3·3)	480 (3·0)	8 (2·5)	7 (2·9)

[a] Percentages are weighted using complex survey weights. Counts are presented unweighted. Information presented is prior to imputation.
[b] Cases from sites which contributed data for ≤3 seasons, were hospital onset cases, or were not sampled for complete chart review were excluded.
[c] Only captured in 2011-2012 season and onward; 4992 observations from 2010-2011 excluded.
[d] Only captured for persons ≥2 years old; 5070 observations from children <2 years old excluded.

Supplemental table 2. Characteristics of hospitalized individuals with influenza A by known versus unknown influenza A subtype (before imputation)

Characteristics	Known subtype n (row %)[a]	Unknown subtype n (row %)[a]	*p*-value[b]
Total	41162 (46·6)	45246 (53·4)	–
Age			<0·0001
0–4 years	2215 (50·1)	2202 (49·9)	
5–17 years	2142 (56·2)	1672 (43·8)	
18–49 years	7621 (47·9)	8299 (52·1)	
50–64 years	9308 (48·1)	9787 (51·9)	
65–74 years	6559 (44·7)	7491 (55·3)	
75–84 years	7035 (44·8)	8130 (55·2)	
≥85 years	6282 (44·0)	7665 (56·0)	
Sex			0·015
Male	19128 (47·1)	20555 (52·9)	
Female	22034 (46·2)	24691 (53·8)	
Race-ethnicity[c]			<0·0001
White, non-Hispanic	24262 (47·1)	26197 (52·9)	
Black, non-Hispanic	7716 (44·2)	9291 (55·8)	
American Indian/Alaskan Native, non-Hispanic	249 (42·1)	349 (57·9)	
Asian/Pacific Islander, non-Hispanic	1654 (40·4)	2190 (59·6)	
Multiracial, non-Hispanic	129 (44·2)	154 (55·8)	
Hispanic	3568 (47·0)	3886 (53·0)	
Underlying medical condition[d]			0·30
No comorbid conditions	4584 (47·2)	4989 (52·8)	
≥1 comorbid conditions	36492 (46·6)	40109 (53·4)	
Seasonal influenza vaccination status[e]			<0·0001
No	17492 (47·9)	18278 (52·1)	
Yes	18769 (46·1)	21061 (53·9)	
Influenza season			<0·0001
2010-2011	2491 (60·6)	1619 (39·4)	
2011-2012	1197 (62·2)	727 (37·8)	
2012-2013	3886 (45·9)	4585 (54·1)	
2013-2014	4783 (60·3)	3155 (39·7)	
2014-2015	6663 (46·0)	7817 (54·0)	
2015-2016	3331 (53·9)	2852 (46·1)	
2016-2017	6017 (45·8)	7113 (54·2)	
2017-2018	6275 (40·0)	8683 (60·0)	
2018-2019	6519 (41·8)	8695 (58·2)	
Pneumonia			0·20
No	31782 (46·5)	35216 (53·5)	
Yes	9380 (47·0)	10030 (53·0)	
Antiviral treatment			<0·0001
No	6816 (58·5)	4690 (41·5)	
Yes	34346 (44·9)	40556 (55·1)	
ICU admission[f]			<0·0001
No	33491 (45·8)	38017 (54·2)	
Yes	7467 (50·9)	6963 (49·1)	
Mechanical ventilation/ECMO use[g]			<0·0001
No	37828 (46·2)	42313 (53·8)	
Yes	3050 (53·5)	2613 (46·5)	
Death[h]			0·014
No	39773 (46·5)	43774 (53·5)	
Yes	1259 (49·0)	1310 (51·0)	

Abbreviations: ECMO, extracorporeal membrane oxygenation; ICU, intensive care unit; IQR, interquartile range

[a] Displays original, unimputed counts. Counts are presented unweighted. Percentages are weighted using complex survey weights. All observations exclude infants <6 months old, those co-infected with multiple influenza types, nosocomial cases, or cases not sampled for complete chart review. Influenza B infections are excluded.

[b] Chi-squared test with Rao and Scott's second-order correction

[c] 7090 (8·2%) observations were missing race and ethnicity information and excluded from the table. While this covariate was not included in the main analysis model, its missing observations were later imputed in the imputation model as part of the chained equations approach.

[d] 273 (0·3%) observations were missing underlying medical condition information and excluded from the table. These missing observations were later imputed in the imputation model.

[e] 11943 (13·8%) observations that were missing seasonal influenza vaccine status and were excluded from the table. These missing observations were later imputed in the imputation model.
[f] 520 (0·6%) observations that were missing ICU status and excluded from the table. These missing observations were later imputed in the imputation model.
[g] 668 (0·8%) observations that were missing mechanical ventilation or ECMO status and were excluded from the table. These missing observations were later imputed in the imputation model.
[h] 307 (0·4%) observations that were missing death status and excluded from the table. These missing observations were later imputed in the imputation model.

Supplemental table 3. Full model of adjusted association between influenza type and imputed subtype and odds of severe in-hospital outcomes

Individual characteristics	ICU admission	Mechanical ventilation/ECMO use	Death
	aOR (95% CI)[a]	aOR (95% CI)[a]	aOR (95% CI)[a]
**Main association**			
Virus type and subtype			
A(H3N2)	Ref	Ref	Ref
A(H1N1)pdm09	1·42 (1·32, 1·52)	1·79 (1·60, 2·00)	1·25 (1·07, 1·46)
Influenza B	1·06 (1·01, 1·12)	1·14 (1·05, 1·24)	1·18 (1·07, 1·31)
**Additional covariates included in the model**			
Age			
6 months─17 years	Ref	Ref	Ref
18─49 years	0·75 (0·70, 0·80)	1·29 (1·17, 1·42)	2·40 (1·83, 3·15)
50─64 years	0·87 (0·82, 0·92)	1·60 (1·45, 1·76)	4·47 (3·45, 5·79)
≥65 years	0·68 (0·64, 0·71)	1·13 (1·02, 1·24)	7·00 (5·44, 9·02)
Seasonal influenza vaccination status			
No	Ref	Ref	Ref
Yes	0·94 (0·91, 0·98)	0·83 (0·79, 0·89)	0·91 (0·84, 0·99)
Influenza season			
2010-2011	Ref	Ref	Ref
2011-2012	0·91 (0·79, 1·04)	0·75 (0·61, 0·92)	0·69 (0·49, 0·96)
2012-2013	1·01 (0·92, 1·11)	0·90 (0·79, 1·04)	0·75 (0·61, 0·93)
2013-2014	1·06 (0·96, 1·17)	0·96 (0·84, 1·10)	0·94 (0·76, 1·17)
2014-2015	0·96 (0·88, 1·05)	0·94 (0·82, 1·07)	0·83 (0·68, 1·01)
2015-2016	0·94 (0·85, 1·04)	0·76 (0·66, 0·87)	0·73 (0·58, 0·91)
2016-2017	1·00 (0·91, 1·09)	0·78 (0·68, 0·89)	0·79 (0·65, 0·96)
2017-2018	0·93 (0·85, 1·01)	0·72 (0·63, 0·81)	0·74 (0·62, 0·89)
2018-2019	0·94 (0·86, 1·03)	0·65 (0·58, 0·75)	0·72 (0·59, 0·87)
FluSurv-NET site			
California	Ref	Ref	Ref
Colorado	1·27 (1·18, 1·36)	1·02 (0·91, 1·14)	0·73 (0·63, 0·85)
Connecticut	0·85 (0·78, 0·93)	0·96 (0·84, 1·09)	0·73 (0·62, 0·87)
Georgia	0·98 (0·90, 1·07)	0·94 (0·82, 1·07)	0·68 (0·58, 0·80)
Maryland	1·05 (0·98, 1·14)	1·12 (1·01, 1·25)	0·89 (0·78, 1·03)
Michigan	1·08 (0·98, 1·19)	1·13 (0·97, 1·31)	0·74 (0·60, 0·92)
Minnesota	0·86 (0·80, 0·93)	0·91 (0·81, 1·03)	0·76 (0·66, 0·88)
New Mexico	0·96 (0·86, 1·07)	1·23 (1·05, 1·43)	0·97 (0·80, 1·18)
New York Albany	0·95 (0·85, 1·05)	1·12 (0·96, 1·31)	0·89 (0·75, 1·07)
New York Rochester	0·70 (0·64, 0·77)	0·92 (0·81, 1·05)	0·70 (0·59, 0·83)
Ohio	1·22 (1·12, 1·34)	1·52 (1·34, 1·73)	0·91 (0·78, 1·07)
Oregon	1·05 (0·96, 1·15)	1·26 (1·10, 1·44)	1·01 (0·87, 1·18)
Tennessee	1·20 (1·10, 1·31)	1·05 (0·92, 1·20)	0·77 (0·64, 0·92)
Utah	1·18 (1·08, 1·30)	0·95 (0·82, 1·10)	0·80 (0·65, 0·98)

Abbreviations: adjusted odds ratio, aOR; confidence interval, CI; ECMO, extracorporeal membrane oxygenation; ICU, intensive care unit

[a] Odds ratios and corresponding 95% confidence intervals were produced using a logistic regression model accounting for survey weights and imputation. The model included influenza type and subtype, age (6 months─17 years, 18─49 years, 50─64 years, ≥65 years), seasonal influenza vaccination status, influenza season, and FluSurv-NET site. The model was rerun for each measure of influenza severity.

Supplemental table 4. Adjusted association between influenza type and imputed subtype and odds of severe in-hospital outcomes adding additional covariates to the multivariable model

Individual characteristics	ICU admission	Mechanical ventilation/ECMO use	Death
	aOR (95% CI)	aOR (95% CI)	aOR (95% CI)
**Original main association[a]**			
Virus type and subtype			
A(H3N2)	Ref	Ref	Ref
A(H1N1)pdm09	1·42 (1·32, 1·52)	1·79 (1·60, 2·00)	1·25 (1·07, 1·46)
Influenza B	1·06 (1·01, 1·12)	1·14 (1·05, 1·24)	1·18 (1·07, 1·31)
**Association also adjusted for underlying medical condition[b]**			
Virus type and subtype			
A(H3N2)	Ref	Ref	Ref
A(H1N1)pdm09	1·42 (1·33, 1·52)	1·79 (1·60, 2·00)	1·25 (1·08, 1·46)
Influenza B	1·07 (1·02, 1·13)	1·15 (1·06, 1·24)	1·18 (1·07, 1·31)
**Association also adjusted for antiviral use[c]**			
Virus type and subtype			
A(H3N2)	Ref	Ref	Ref
A(H1N1)pdm09	1·41 (1·32, 1·51)	1·78 (1·59, 1·99)	1·26 (1·08, 1·47)
Influenza B	1·08 (1·02, 1·14)	1·16 (1·07, 1·25)	1·15 (1·04, 1·28)

Abbreviations: adjusted odds ratio, aOR; confidence interval, CI; ECMO, extracorporeal membrane oxygenation; ICU, intensive care unit

[a] Odds ratios and corresponding 95% confidence intervals were produced using logistic regression accounting for survey weights. The model included influenza type and subtype, age (6 months─17 years, 18─49 years, 50─64 years, ≥65 years) seasonal influenza vaccination status, influenza season, and FluSurv-NET site.

[b] Odds ratios and corresponding 95% confidence intervals were produced using logistic regression accounting for survey weights. The model included influenza type and subtype, age (6 months─17 years, 18─49 years, 50─64 years, ≥65 years), seasonal influenza vaccination status, influenza season, FluSurv-NET site, and presence of an underlying medical condition.

[c] Odds ratios and corresponding 95% confidence intervals were produced using logistic regression accounting for survey weights. The model included influenza type and subtype, age (6 months─17 years, 18─49 years, 50─64 years, ≥65 years), seasonal influenza vaccination status, influenza season, FluSurv-NET site, and antiviral use.

Supplemental table 5. Adjusted odds ratios of severe in-hospital outcomes by influenza type and imputed subtype stratified by age and additionally adjusted for underlying medical conditions

	ICU admission		Mechanical ventilation/ECMO use		Death	
	**A(H1N1)pdm09 *vs.* A(H3N2) aOR (95% CI)[a]**	**B *vs.* A(H3N2) aOR (95% CI)[a]**	**A(H1N1)pdm09 *vs.* A(H3N2) aOR (95% CI)[a]**	**B *vs.* A(H3N2) aOR (95% CI)[a]**	**A(H1N1)pdm09 *vs.* A(H3N2) aOR (95% CI)[a]**	**B *vs.* A(H3N2) aOR (95% CI)[a]**
Overall	1.42 (1·32, 1·52)	1·06 (1·01, 1·12)	1·79 (1·60, 2·00)	1·14 (1·05, 1·24)	1·25 (1·07, 1·46)	1·18 (1·07, 1·31)
Age						
6 months–<5 years	1·32 (1·06, 1·64)	1·00 (0·84, 1·19)	1·57 (1·08, 2·28)	1·36 (1·00, 1·84)	0·91 (0·28, 2·98)	1·39 (0·54, 3·58)
5–17 years	1·30 (1·04, 1·62)	0·88 (0·74, 1·04)	1·87 (1·23, 2·85)	1·24 (0·91, 1·68)	1·81 (0·48, 6·91)	2·87 (1·32, 6·26)
18–49 years	1·43 (1·25, 1·62)	1·12 (1·00, 1·25)	1·78 (1·46, 2·17)	1·24 (1·04, 1·48)	1·84 (1·17, 2·92)	1·41 (0·95, 2·08)
50–64 years	1·44 (1·26, 1·65)	1·06 (0·96, 1·18)	1·78 (1·47, 2·14)	1·09 (0·94, 1·27)	1·50 (1·14, 1·98)	1·12 (0·88, 1·42)
65–74 years	1·40 (1·16, 1·68)	1·04 (0·91, 1·19)	1·62 (1·25, 2·11)	1·02 (0·84, 1·24)	1·44 (1·03, 2·03)	1·23 (0·97, 1·56)
75–84 years	1·36 (1·12, 1·67)	1·17 (1·01, 1·34)	1·46 (1·08, 1·98)	1·12 (0·91, 1·39)	1·14 (0·83, 1·57)	1·33 (1·09, 1·63)
≥85 years	1·23 (0·91, 1·68)	1·18 (1·01, 1·39)	1·56 (0·95, 2·57)	1·16 (0·88, 1·53)	1·05 (0·70, 1·59)	1·10 (0·92, 1·33)

Abbreviations: adjusted odds ratio, aOR; confidence interval, CI; ECMO, extracorporeal membrane oxygenation; ICU, intensive care unit

[a] Odds ratios and corresponding 95% confidence intervals were produced using logistic regression models accounting for survey weights. The model included influenza type and subtype, seasonal influenza vaccination status, influenza season, FluSurv-NET site, presence of an underlying medical condition.

Supplemental table 6. Adjusted association between influenza type and subtype and odds of severe in-hospital outcomes using original, unimputed data set that excluded observations missing influenza A subtype

	ICU admission		Mechanical ventilation/ECMO use		Death	
	**A(H1N1)pdm09 *vs.* A(H3N2) aOR (95% CI)[a]**	**B *vs.* A(H3N2) aOR (95% CI)[a]**	**A(H1N1)pdm09 *vs.* A(H3N2) aOR (95% CI)[a]**	**B *vs.* A(H3N2) aOR (95% CI)[a]**	**A(H1N1)pdm09 *vs.* A(H3N2) aOR (95% CI)[a]**	**B *vs.* A(H3N2) aOR (95% CI)[a]**
Overall	1.40 (1·29, 1·51)	0·95 (0·89, 1·01)	1·77 (1·58, 1·99)	0·99 (0·90, 1·09)	1·30 (1·10, 1·54)	1·14 (1·00, 1·30)
Age						
6 months─17 years	1.20 (1.00, 1.44)	0.79 (0.68, 0.91)	1.67 (1.22, 2.30)	1.01 (0.78, 1.31)	1.32 (0.48, 3.63)	1.76 (0.85, 3.65)
18─49 years	1.46 (1.24, 1.72)	0.99 (0.85, 1.14)	1.99 (1.56, 2.53)	1.21 (0.97, 1.51)	2.38 (1.38, 4.12)	1.74 (1.02, 2.97)
50─64 years	1.36 (1.18, 1.57)	0.90 (0.79, 1.02)	1.78 (1.45, 2.18)	0.94 (0.78, 1.13)	1.33 (0.95, 1.87)	0.94 (0.68, 1.30)
≥65 years	1.49 (1.29, 1.71)	1.03 (0.94, 1.14)	1.73 (1.38, 2.17)	0.93 (0.80, 1.09)	1.07 (0.84, 1.36)	1.13 (0.97, 1.32)
Seasonal influenza vaccination status						
No	1.42 (1.27, 1.60)	0.97 (0.88, 1.05)	1.56 (1.30, 1.87)	0.94 (0.82, 1.08)	1.04 (0.80, 1.35)	1.08 (0.91, 1.28)
Yes	1.37 (1.23, 1.52)	0.94 (0.86, 1.02)	1.95 (1.67, 2.28)	1.06 (0.93, 1.21)	1.61 (1.26, 2.05)	1.24 (1.01, 1.51)

Abbreviations: adjusted odds ratio, aOR; confidence interval, CI; ECMO, extracorporeal membrane oxygenation; ICU, intensive care unit

[a] Odds ratios and corresponding 95% confidence intervals were produced using logistic regression models accounting for survey weights. The model included influenza type and subtype, age (6 months─17 years, 18─49 years, 50─64 years, ≥65 years), seasonal influenza vaccination status, influenza season, and FluSurv-NET site.

Supplemental table 7. Comparison of influenza A subtype proportions before and after imputation by influenza season

Season	Virus	Before imputation (%)	After imputation (%)[a]
2010-11	A(H3N2)	63.9	64.1
	A(H1N1)pdm09	36.1	35.9
2011-12	A(H3N2)	75.3	76.3
	A(H1N1)pdm09	24.7	23.7
2012-13	A(H3N2)	95.9	96.0
	A(H1N1)pdm09	4.1	4.0
2013-14	A(H3N2)	9.3	9.8
	A(H1N1)pdm09	90.7	90.2
2014-15	A(H3N2)	99.8	99.7
	A(H1N1)pdm09	0.2	0.3
2015-16	A(H3N2)	12.6	12.7
	A(H1N1)pdm09	87.4	87.3
2016-17	A(H3N2)	98.1	98.0
	A(H1N1)pdm09	1.9	2.0
2017-18	A(H3N2)	83.0	82.4
	A(H1N1)pdm09	17.0	17.6
2018-19	A(H3N2)	46.3	46.9
	A(H1N1)pdm09	53.7	53.1

[a] Percentage presented is a mean percentage of each virus subtype averaging across the 30 imputed data sets.

Supplemental figure

Supplemental figure 1. Directed acyclic graph of relationship between influenza type and subtype and the association with severe in-hospital outcomes. This figure illustrates causal pathways in the relationship between infection with influenza virus type and subtype and severe in-hospital outcomes. The minimal sufficient adjustment set for the association between influenza virus type/subtype and severe influenza-associated outcomes was age, state of residence, seasonal influenza vaccination, and influenza season. Variables not captured in the analysis data set are indicated with dashed lines.

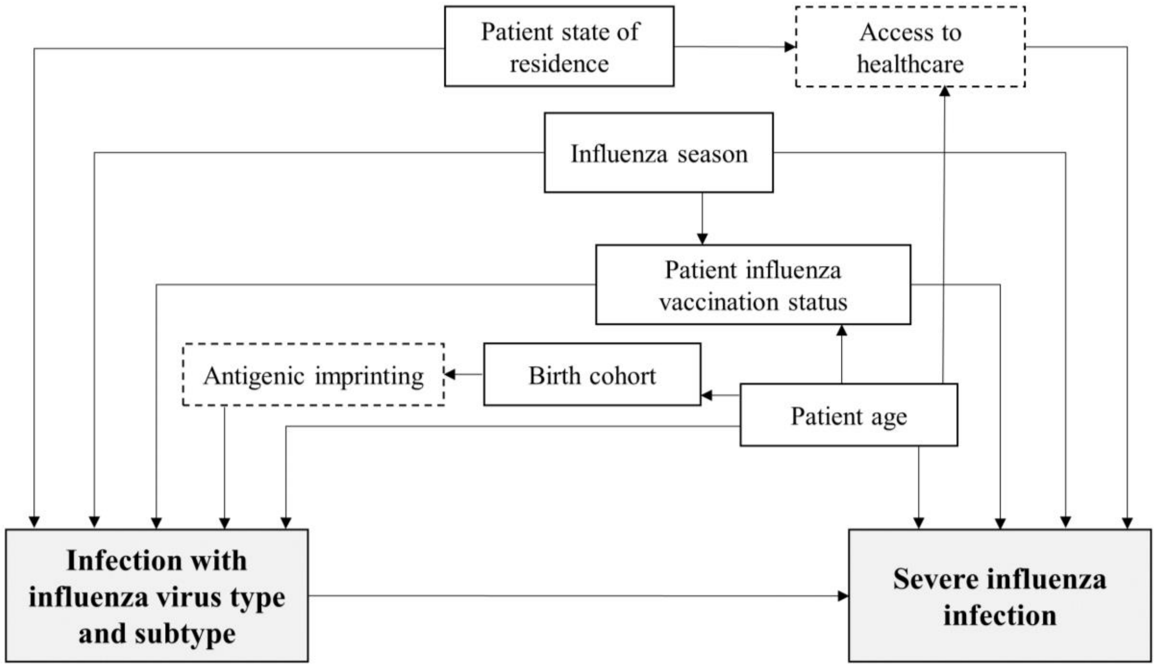

Supplement: 1 [file NIHMS1942263-supplement-1.pdf]
